# Removal of a Tungsten Carbide Ring from the Finger of a Pregnant Patient: A Case Report Involving 2 Emergency Departments and the Internet

**DOI:** 10.1155/2016/8164524

**Published:** 2016-03-06

**Authors:** Alexandre Moser, Aristomenis Exadaktylos, Alexander Radke

**Affiliations:** ^1^Department of Surgery, Spital Zweisimmen, Spital STS AG, Karl Haueter-Strasse 21, 3770 Zweisimmen, Switzerland; ^2^Department of Emergency Medicine, Inselspital, University of Bern, Freiburgstrasse, 3010 Bern, Switzerland

## Abstract

*Introduction*. Destructive or nondestructive procedures may be used to remove rings from injured fingers. Because of their hardness, tungsten carbide rings present special problems.* Case Presentation*. The patient was a 33-year-old woman, two weeks before delivery, with a swollen and reddened ring finger. It was decided to remove a tungsten carbide ring from her ring finger. This was achieved by shattering the ring with locking pliers. The patient's ring finger recovered fully.

## 1. Introduction

The most common reason for ring removal is swelling, when rings can have a painful tourniquet effect on digits, thereby causing ischemia. This can be exacerbated by soft tissue injuries, dislocations, fractures, or allergic reactions or anaphylaxis [1]. Swollen fingers may also be associated with pregnancy.

Nondestructive removal of the ring is always preferred, as it may be of great sentimental value to the patient. However, this is not always possible if the swelling is severe. It may be necessary to cut through the ring.

In such a case, tungsten carbide rings present a special problem, as they are extremely hard.

On the other hand, these rings have very low flexibility and model studies show that they may be shattered with locking pliers [2, 3]. This approach has now been applied clinically to a patient shortly before delivery.

## 2. Case Presentation

**Figure 1:**
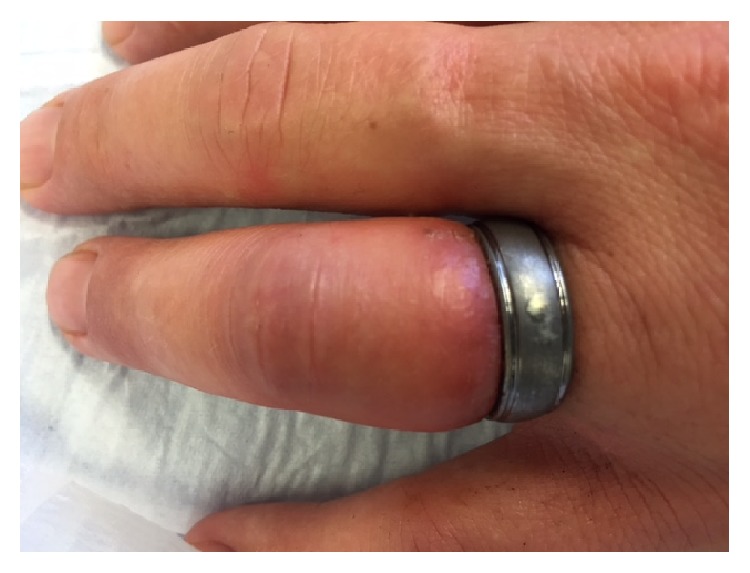
Patient's hand on presentation.

The patient presented to the emergency room of a rural hospital with a tungsten carbide ring on a highly swollen and painful ring finger. Capillary refill was prolonged with concomitant superficial lacerations (Figure 1). The patient's primary physician had already unsuccessfully attempted to remove the ring using conventional methods. The staff of the emergency room then tried to remove the ring by cutting, but again without success.

A consultation was sought from a university hospital ED and transfer was considered because of the finger threatening. The attending university hospital ED physician also lacked experience in this kind of material and therefore the Internet was consulted simultaneously which led to a Youtube video [4]. The technique was applied successfully as described below. It was then unnecessary to transfer the patient.

**Figure 2:**
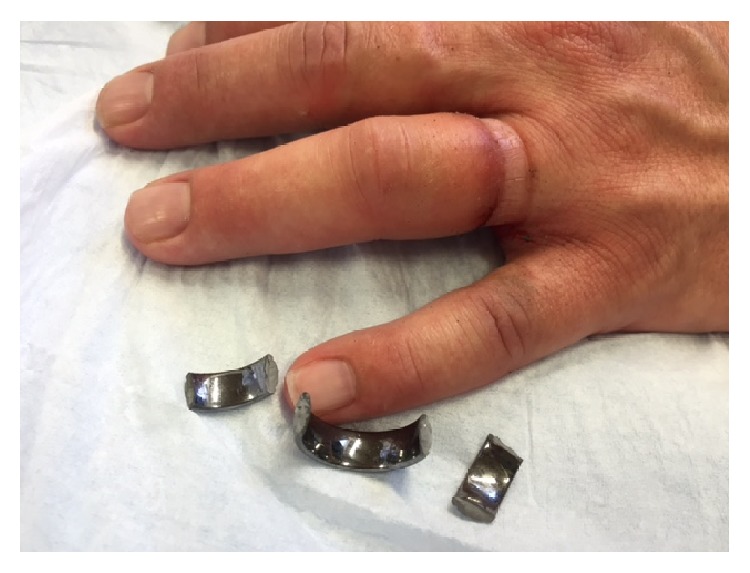
Patient's hand and ring after removing the ring.

The jaws of the locking pliers were adjusted so that when closed they gripped the ring snugly without being excessively tight. They were then opened and the tightening screw was turned one-quarter of a turn to the right to slightly increase the grip pressure on the ring. The pressure was then reapplied and the process was repeated until the ring was seen, heard, or felt to fracture. The finger was not squeezed during this procedure.  Figure 2 shows the resulting ring fragments and the appearance of the finger after the ring had been removed.

## 3. Discussion

According to the algorithm described by Kalkan et al. [5], ring removal was mandatory in this case and the material of the ring was known. Consultation of the university hospital ED led to the locking pliers technique.

Allen et al. [2] applied the locking pliers technique to the removal of tungsten carbide rings from cadaveric fingers. Finger swelling was induced by injecting a fluorescein and saline solution. Six rings were successfully removed. Superficial lacerations were recorded in two fingers, but no phalangeal fractures.

Gardiner et al. [3] removed tungsten carbide rings from mannequin's fingers, using either the locking pliers technique or the umbilical tape technique. Distal edema was simulated using foam tape. The locked pliers technique was quicker (mean 23.1 sec [95% CI 15.4–30.8] versus 135.4 sec [95% CI 130.2–150.6]). However, the locking pliers technique destroyed all rings and caused sharp ring fragments to be thrown up to 70 cm. This was not a problem in the present case.

The present case is the only clinical application of this procedure of which we are aware.

The ring was rapidly removed within 30 seconds and clinical recovery was complete.
